# Editorial: “Neuromodulation of Language, Cognition and Emotion”

**DOI:** 10.3390/brainsci12020136

**Published:** 2022-01-20

**Authors:** Sara Borgomaneri, Manuel de Vega

**Affiliations:** 1 Centro Studi e Ricerche in Neuroscienze Cognitive, Dipartimento di Psicologia, Campus di Cesena, Alma Mater Studiorum-Università di Bologna, 47521 Cesena, Italy; 2 IRCCS Fondazione Santa Lucia, 00179 Rome, Italy; 3 Instituto Universitario de Neurociencia (IUNE), Universidad de La Laguna, 38200 Santa Cruz de Tenerife, Spain

Neuromodulation can be defined as the alteration of brain activity by delivering physical stimuli to a specific neural region. This Special Issue of *Brain Sciences* is primarily concerned with one type of neuromodulation: non-invasive brain stimulation (herein, NIBS), which can be achieved through the administration of magnetic pulses (Transcranial Magnetic Stimulation, or TMS) and through electrical stimulation (Transcranial Direct Current Stimulation, or tDCS). The use of such NIBS techniques for neuroscientific research has grown exponentially in recent years, since they allow researchers to estimate causal links between brain and functions, beyond the correlational data provided by other brain recording techniques. In addition, NIBS protocols are employed for the treatment of neurological and psychiatric disorders. In the present Special Issue, we have invited authors with expertise in neuromodulation methods to submit original manuscripts aimed at mapping brain functions, in a broad sense, or at the diagnosis, therapy and rehabilitation of neural or psychopathological disorders. The result is a nice set of articles involving a mix of techniques and stimulation protocols, which range from studies of neurocognitive or emotional functions in healthy participants to intervention studies with patients.

As a technique, TMS can be used to momentarily perturb brain functioning and measure the physiological or behavioral consequences, which allows making causal inferences about the involvement of brain areas in cognitive processes. It is possible to deliver TMS in several ways, from single-pulse TMS methods up to pair-pulse and more complex multi-pulse paradigms, employed for different purposes. Thus, while single-pulse and paired-pulse TMS protocols are mainly used to map brain functions, repetitive TMS (rTMS) is used to induce changes in brain activity that can last beyond the stimulation period. Single pulse TMS can be used to probe the chronometry of basic processes within the brain and, combined with electromyography (EMG), to probe changes in the excitability of the corticospinal system. Indeed, administering single pulses over the primary motor cortex (M1) leads to a twitch in the target muscle, evoking motor-evoked potential (MEP) recorded by EMG. The amplitude of MEPs is usually used to assess the corticospinal tract excitability in a precise time-point. On its side, rTMS applied at certain frequencies or burst intervals can induce relatively long-lasting changes in plasticity of the target brain region, which in turn cause changes in the excitability of the cortex and eventually in the associated functions. The changes in excitability have been linked to long-term potentiation and long-term depression, which are thought to underly an increase or decrease in the likelihood of action potentials taking place, respectively. The persistent effects of rTMS makes theses protocols useful not only for the mapping of brain functions but also for therapeutical interventions.

An example of how single-pulse TMS can be used to investigate the changes in motor excitability comes from the study from Borgomaneri and colleagues [1], who investigate the possible modulations in corticospinal excitability while healthy participants observed happy, fearful and neutral pictures of facial expressions. The right and left primary M1 excitability was assessed at 150 and 300 ms after picture onset. The results showed an early increase in the right M1 excitability (i.e., higher MEPs amplitude) for the observation of happy and fearful emotional faces compared to neutral expressions, which was predicted by interindividual differences in the disposition to experience aversive feelings (personal distress) in interpersonal emotional contexts. No differences in corticospinal excitability were observed at the later time (300 ms) or in the left M1. These findings demonstrated that single-pulse TMS is a useful tool to map the time course of M1 involvement during emotion perception, highlighting the role of the right hemisphere in implementing a rapid and transient facilitatory response to emotionally arousing stimuli, such as emotional facial expressions. Interestingly, although photos are static pictures, they evoke similar neural responses in the action observation network (AON) as live actions or video clips of actions. In his review, Kislinger [2] presents studies on neural responses to pictured actions in the AON and the cognitive functions of these responses. He reports TMS studies, which suggest that seeing action photos conveys similar processes of social perception as observing live actions. The high temporal resolution of the TMS provides accurate information on the timing of such action-specific neural representations, which begin around 150 ms after stimulus onset and lasted until around 300 ms. Moreover, TMS offers information related to the specific contributions of cortical regions to the perception and understanding of actions, as well as causal links between these regions. The reviewed studies showed that interfering with the activity in somatosensory cortices impaired the perception and recognition of specific properties of postures or movements of body parts or of properties of the objects involved in actions. Thus, TMS studies provided decisive information about the crucial role of these cortices in the perception and understanding of observed actions. Kislinger concluded that such effects indicate that the neural representations of action elements that are established in the AON are more specific and comprehensive than would be the case with mere categorization.

In a similar way, dual-site TMS can be successfully used for non-invasively mapping causal connectivity between two interconnected brain regions with high temporal resolution. Breveglieri and coworkers [3] used the paired-pulse TMS to investigate the time-course of the functional connectivity between the left medial posterior parietal cortex (PPC) and the left M1 in healthy participants while they remained at rest. The results showed that the PPC exerts a significant inhibition over M1 when the inter-stimulus intervals (ISIs) were longer than 10 ms, suggesting that these modulations may reflect the activation of different parieto-frontal pathways, with long latency inhibitions likely recruiting polysynaptic pathways, presumably through anterolateral PPC. On the other hand, the rTMS protocols can be employed for disrupting the functional integrity of a target brain region and thus assessing its role in a given process.

Pavan at al. [4] used the rTMS protocol for interfering with the activity of the human medio-temporal complex (hMT+) in order to investigate its crucial role in in the memory encoding of visual motion direction. Healthy participants were asked to indicate whether a test random dot kinematogram (RDK) had the same motion direction as any of the four RDKs shown in the memory sample. RTMS was delivered either in an early (encoding) or late (retention) phase during the retention interval and after the offset of the rapid motion sequence. Additionally, in a second experiment, it was assessed whether rTMS interferes with the precision of the memory trace for motion direction and serial position in the temporal sequence. The results showed that rTMS delivered during the early (but not the late) phase of the retention interval hampers not only the recognition of the RDKs presented in middle positions (i.e., difficult to recognize) but also the precision of the retained motion direction. Moreover, the results from experiment 2 revealed a disruption of precision performance when rTMS was delivered over hMT+ and only for RDKs in the middle position, suggesting that the functional integrity of the hMT+ is crucial not only for the recognition but also the precision of the stored visual information.

Importantly, neuromodulation can be also used for improving a brain function. Anodal tDCS is a non-invasive brain stimulation tool which, similar to rTMS, although less focally, induces long-term potentiation (LTP) plasticity of the human brain via application of weak direct currents. Anodal tDCS was applied by Reyes and colleagues [5] over the temporal cortices (STS) aiming at improving participant’s reading speed. The results showed that anodal tDCS had significant effects in reading speed for social and neutral sentences in participants with low scores in the behavioral approach system (BAS) and the behavioral inhibition system (BIS) scales. This result nicely demonstrated that cognitive abilities can be improved by neurostimulation and that the effects can be modulated by approach and avoidance personality traits.

On another side, neuromodulation can be used in several clinical domains. In the present topic, we collected some examples showing that NIBS protocols can be effective treatments by themselves or in combination with classical rehabilitation protocols in several neurological and psychiatric diseases. Ahorsu and colleagues [6] provided the first systematic review aiming to evaluate the therapeutic efficacy of NIBS (tDCS and TMS) on cognitive functions among people with traumatic brain injury (TBI). Overall, the results of nine studies showed that the impact of the NIBS protocols on cognition in people with TBI was moderately significant with very low heterogeneity across studies. Specifically, significant and marginally significant moderate effect sizes were found for cognitive sub-domains including attention, memory and executive function. These findings suggested that NIBS is moderately effective in improving cognitive functions among people with TBI. In particular, NIBS may be used as an alternative and/or complementary treatment to the traditional approach in rehabilitating cognitive functions in people with TBI.

In the attempt to improve action semantic skills in Parkinson’s disease (PD) patients, Suárez-García and her colleagues [7] investigate the ability of the anodal tDCS over the primary motor cortex, combined with cognitive training, to ameliorate action–concept processing. On day 1, 22 PD patients were requested to complete a picture–word association (PWA) task involving action-verb and object-noun conditions. Subsequently, they were asked to perform online PWA practice over three days while they were stimulated with anodal tDCS or with sham mode. Finally, at day 5, the initial protocol was repeated. The anodal tDCS group exhibited faster reaction times for action (as opposed to object) concepts in the post-stimulation test, suggesting that action–concept deficits in PD are distinctively grounded in motor networks and might be countered by direct neuromodulation of such circuits. Another example which demonstrated the efficacy of the anodal tDCS in improving cognitive functions comes from Padrón and colleagues [8], who addressed the possibility of improving mentalizing processes in adults with autistic traits. To achieve this goal, the author applied the autism quotient scale (AQ) to a large sample of university students and recruited those with high-AQ scores. They initially received sham tDCS before completing a pre-test in two mentalizing tasks: false belief and self-other judgments. Over the next week, on four consecutive days, they received sessions of anodal tDCS over the right temporo-parietal junction (rTPJ), a region frequently associated with the theory of mind. On the last day, they completed a new set of mentalizing tasks. A control group (with low-AQ scores) matched in age, education and intelligence received just sham stimulation and completed the same pre-test and post-test. The results showed that the high-AQ group selectively improved their performance (faster responses) after anodal tDCS in the false belief and in the self–other judgments of mental features, whereas they did not change performance in the control tasks of equivalent complexity, such as false photographs or the self–other judgments of physical features.

In conclusion, the articles collected in this Special Issue are representative of ongoing research in the dynamic field of non-invasive neuromodulation. The reader could have a glimpse of how advanced NIBS protocols are being applied to the study of brain functions in healthy individuals as well as neural rehabilitation and therapy in psychopathological conditions. Furthermore, the reader could also share the excitement for new questions, technical developments and research opportunities that are still emerging in the field.
